# Attitudes of Iranian students about organ donation: a qualitative study

**DOI:** 10.1186/s12910-019-0372-z

**Published:** 2019-05-28

**Authors:** Parisa Parsa, Malihe Taheri, Forouzan Rezapur-Shahkolai, Samane Shirahmadi

**Affiliations:** 1 Chronic Diseases (Home Care) Research Center, Hamadan University of Medical Science, Hamadan, Iran; 2 Department of Public Health, School of Health and student Research Center, Hamadan University of Medical Sciences, Hamadan, Iran; 3 Department of Public Health, School of Public Health and Research Center for Health Sciences, Hamadan University of Medical Sciences, Hamadan, Iran

**Keywords:** Brain death, Tissue and organ procurement, Student, Attitude, Interview

## Abstract

**Background:**

Organ donation is a life-saving process for patients suffering from an advanced organ failure.

A disparity between donated organs and required organs for transplantation is one of the major problems in Iran. Since personal attitudes about organ donation is a main factor influencing willingness to donate organ, the present study sought to provide a deeper understanding of the attitudes of university students in Iran regarding organ donation.

**Methods:**

This qualitative study was conducted in 2016. Semi-structured interviews were held for collecting data from eighty five students from various universities in Hamadan city, Iran. Using a purposive sampling method, the students were selected based on the maximum variation. The content analysis method was used for data analysis by the research team and criteria for the study’s rigor was considered.

**Results:**

Overall, the students had positive attitudes toward organ donation by brain-dead patients. Nevertheless, not of them stated that they would become an organ donor. During the data analysis, 376 primary codes, 13 categories, and 6 themes were developed. Themes were “cognitive readiness”, “mediators of decision making”, “beliefs and motivations”, “interactions with the health system”, “dependency”, and “integrity of the body”. Also, thirteen sub-themes were developed.

**Conclusion:**

Many factors influence the students’ attitudes toward organ donation. Identification and explanation of these factors can help healthcare managers and policymakers for planning and improving the organ donation culture in the society.

## Background

Organ transplantation is an effective approach for the treatment of organ failure [1]. During the past three decades, demands for organ transplantation have been increased up to 70% in comparison to the past [2, 3].

One of the main sources of organ supply in the world is patients with brain death [4]. It has been shown that 15–67% of patients with brain death are suitable donors of solid organs [5]. Accordingly, the healthcare system tries to encourage people to donate their organs in case of brain death [6]. Nevertheless, as high as 10–25% of patients waiting for an organ transplantation will die because of the lack of an organ donor [7].

A lack of financial support, responsibility by policy makers, legislations, and bureaucratic issues, shortages of specialist workforces, religious diversities, and the society attitude toward organ donation affect organ donation from a brain-dead person [8–13]. Kim et al., in South Korea reported that attitude, belief, and behavior of people regarding organ donation are connected to the social, cultural, and religious context [1].

Organ transplantation in its modern and present sense in Iran was started in 1935 with a corneal transplantation. The first Iranian kidney transplant was performed in 1968 and the second kidney transplantation center was established in 1985 [14].

Given the increased need for transplantation and the need for heart, lung and liver transplantation, measures to increase organ donation following brain death was required.

Therefore, the organ transplantation bill was submitted to the Islamic Consultative Assembly of Iran in 1993. After several revisions and the advent of Imam Khomeini (the leader and the first Shia jurisprudent in Iran) and his agreement on this, this law was passed in 2000. In 2002, with the establishment of a special affairs and organ transplant department in the Ministry of Health, organ donation among patients with brain death was officially launched [14–16]. In Iran, a patient or their family must pay for healthcare. If a brain-dead person donates organs from a public hospital, healthcare fees are paid by the government; making the patient’s healthcare costs free of charge for the patient’s family.

While global statistics on organ donation by brain-dead patients in countries such as France, Italy and the United States are 20–30 per million populations [17] and in a country like Spain, this figure is close to 43.4 and Croatia is 38 per million population. In spite of efforts made in Iran, the donation rate reached only 10.9 per million population in 2017 and ranked the 26th in the world [18].

In Iran, from a population of 81 million people, 5000–8000 brain deaths occur annually [19]. Potentially 2500–4000 people annually could become organ donors. However, in 2017, only 926 people of those who died of a brain death meaning less than one-third of them agreed to donate organs, of which the number of donations was much lower than that in European and American countries [19]. This has caused a daily death record of 7–10 patients with a need to transplantation in Iran due to a lack of timely transplantation [19].

In terms of transplants per million population in Iran, in 2016 there were 16.7 kidney transplantations, 9.5 liver transplantations, 1.8 heart transplantations, 0.2 pancreas transplantations and 0.2 lung transplantations from brain-dead donors. In terms of worldwide transplantation from brain-dead donors, Iran was ranked 23th, 18th, 28th, 26th and 28th respectively for these organs [18].

The results of previous qualitative studies conducted in Iran have indicated that four groups of factors influence organ donation: for physicians and hospital crews’ perspectives (awareness, attitude, and self-confidence) [20, 21], individuals and their families (awareness and attitude toward organ donation) [22], cultural and regulation issues [23] and socioeconomic issues affect [20, 21]. In contrast, not considering religion religious perspectives, considerations such as unwillingness to part with body organs, family opposition, and the induction of hopelessness by friends and family members are the most important reasons why people refuse to register as an organ donor [24–26].

For designing an effective intervention, there is a need to understand people’s attitudes and assess they affect the process of decision making regarding organ donation after brain death. University students as a well-educated, liberal and influential population have a great influence on the society and their family [27]. A few studies have been conducted to assess the attitudes of students toward organ donation in Iran. In the present qualitative study, students’ attitudes and regarding organ donation in order to plan intervention program for improving the attitude of society toward organ donation.

## Methods

### Ethical considerations

This study was approved by the Ethics Committee of Hamadan University of Medical Sciences (IR.UMSHA.REC.1396.437). The informed consent form was signed by subjects after informing them of the study objectives, risks and benefits, the voluntary nature of participation in the study and recording of their voices.

### Design

Considering the study’s aim, a qualitative content analysis method was used to explore human emotions and perceptions hidden behind their experiences [28]. In the present study, Semi-structured interviews with students from various universities in Hamadan city, Iran were held. Collected data was analyzed, categorized and themes were extracted [29].

### Participants

To recruit appropriate individuals, a purposive sampling method was used with a maximum variation, so that 6 major universities in the city of Hamadan (Bu Ali Sina, Medical Sciences, Islamic Azad University, University of Applied Science and Technology, Shahid Maghsoudi Teacher Training, Hamadan University of Technology) were selected. Next, an advertisement was installed on the billboard located in students’ traffic places to invite them for participation in the study. A research team member was responsible for distributing tracts containing information about the study, type of study, and methods of contacting the researchers. For 1 month at the universities, students who were interested in participating in the study were registered. Appropriate coordination was made with volunteer students to determine the appropriate time and place for interviewing. Each student after the interview, were asked to introduce new students who had a similar situation in terms of attitudes toward organ donation.

Inclusion criteria were being student at Hamadan city and providing informed consent to participate in the study. Exclusion criterion was getting absence from interviews twice even after conducting follow-ups by the researchers.

### Data collection

A total number of ninety interviews in Farsi language were held for data collection. The first five interviews helped find weak and ambiguous points of the interview process. Each interview lasted for 45–60 min.

After determining demographic characteristics and personal experience of organ donation of participants, four structured questions about the reasons why the interviewee tended or did not tend to want to donate his/her organs after becoming brain dead were asked.

“What do you know about brain death?”

“What is your opinion about donating organs by someone who is brain-dead?”

Branching questions were asked to follow their perspectives:

“Could you please explain to me more about this issue?”

“Would you like to donate your useful organs, if you became brain-dead?”

After each interview, the interview audio tapes were immediately transcribed along with their non-verbal communication such as crying, smiling, silence etc. The transcriptions were compared with audio-tapes. The interviews were continued from June 2016 to September 2017. Sampling was continued until data saturation was reached at the 85th interview, but 5 additional interviews were conducted to ensure consistency in data.

The interviews were conducted by two doctoral students in the fields of health education and promotion affiliated with Hamadan University of Medical Sciences. To ensure that the same interview procedure was performed, the interviews were conducted in the presence of both interviewers.

### Data analysis

The data were analyzed using the conventional content analysis method. This method is commonly utilized in inductive qualitative studies aiming at gaining a deeper understanding of a phenomenon. According to this approach, themes and concepts were directly extracted from data gathered during the interviews.

Transcribed interviews by the first author were analyzed using the constant comparative analysis [30]. At the first level of analysis, open source coding was used to group data into codes. Axial coding was made to determine the patterns and features of data. The codes were compared together and similar codes fitted in one category, which were compared together to ensure their distinctions. New data was consistently compared with the analyzed data, which allowed them to be grouped into categories or themes, and to be modified. Through selective coding the central category was determined. Individuals’ quotations supporting categories and themes were identified to ensure reliability of data.

### Rigor

Three methods were used to create accuracy, provide transferability, and minimize the bias in this study. In this study, interviews with different people increased variation in the data in terms of gender, age, and study field, which increased confirm ability, credibility and transferability.

Data accuracy was ensured through the researchers’ integration during the data analysis. Hence, each interview was separately coded by three researchers, and the coding process was compared and discussed by the researchers to reach a consensus. In addition, an expert professor in the field of qualitative research supervised all stages of the study. The allocation of sufficient time and the open and sympathetic relationship with the participants contributed to credibility.

## Results

The mean age of the participants was 30.4 years (SD ± 1.86), 43.3% were studied in medical science and 38.9% were Bachelor/Associate. The demographic characteristics of the research participants were presented in Table 1.

**Table 1:** Student’s characteristics (*n* = 90)

student’s characteristics	Categories	N (%)
Age group (year)	20–30	45 (50)
	31–40	33 (36.6)
	41–50	11 (12.2)
	Over 50	1 (1.11)
Gender	Male	48 (53.3)
	Female	42 (46.6)
Field of Study	Medical science	38(42.2)
	Engineering	31(34.4)
	Humanities	21(23.3)
Grade Level	Associate/Bachelor	35(38.9)
	Master’s Degree	31 (34.5)
	PhD, Doctor of Medicine	24 (26.6)
University	Medical Science	30 (33.3)
	Bu-Ali Sina University	20 (22.2)
	University of Applied Science and Technology	10 (11.1)
	Islamic Azad University	20 (22.2)
	Shahid Maghsoudi Teacher Training	5 (5.55)
	University of Technology	5 (5.55)

The data analysis resulted in identifying 376 primary codes, 13 categories, and 6 themes. Themes were as “cognitive readiness”, “mediators of decision making”, “beliefs and motivations”, “interactions with the healthcare system”, “dependency”, and “integrity of the body” (Table 2).

**Table 2:** Themes, categories and codes, extracted from the study

Relevant theme derived from categories	Relevant categories	Examples of code
Cognitive readiness	- Media influence - knowledge and awareness - Previous experience	- The impact of video and TV series on the public’s attitude about organ donation, emphasizing the scientific aspect of it in the media rather than stimulating sentimental emotions; - Increasing public awareness about brain death and donating organs to facilitate the donation process at the time of brain death, rectification of false religious beliefs; - The effect of understanding people’s knowledge and their living conditions, effect of family members’ illness on their willingness to donate organs.
Mediators of decision making	- Family consent - Characteristics of the brain-dead patient	- Adequacy of the family’s knowledge of the individual’s decision to donate organs, the consent during lifetime for organ donation in case of brain death, - Uncertainty about the logic of the deceased’s decision for organ donation during life time, the degree of relationship with the deceased, the commitment to the will of the dead person, lack of consideration of the issue of organ donation by relatives because of its suffering identity, the hope for returning to the life,
Beliefs and motivations	- Internal beliefs and motivations - Religious beliefs	- The sense of benevolence, giving life to others, reducing the suffering of others. - Contributing to the advancement of science, retaining a good memory of the person after death, loss of organs after burial and usability of organs in the body, belief in the mutilation of the body, accountability of the donor toward sin actions by the recipient, giving no values to the life of other people, not wanting to mix bodies. - Belief in the physical resurrection of organ donation, oppressing the body in case of organ donation, suffering the soul with organ donation. - Interference in God’s affairs, believe in predestination, the chance of passing bad characteristics of the organ donor to the receiver.
Interactions with the healthcare system	- Medical errors - The lack of trust to the healthcare services	- Diagnosis of brain death by the physician, shortages of specialists and adequate equipment, inappropriate treatment of relatives of the deceased by medical staff - The lack of a system for organ donation and transplantation, not giving enough time to decide on organ donation, lack of a specific organization to prevent abuse of transplantation.
Dependency	- Acceptance - Surrender	- Feeling saddened by the perception of brain death in the first degree relatives - Unwillingness to think of brain death of the first-degree relatives and donating membership.
Integrity of the body	- Fear of being slaughtered - Feeling of being guilty	- An unpleasant impression of the torn fragmentation of the body after death, the feeling of belonging to organs. - Feeling of conscientiousness in the family of donor due to body defect, discomfort from torn body of loved ones.

### Theme 1: Cognitive readiness

This theme was consisted of three categories as media influence, knowledge and awareness, and previous experience regarding organ donation. Most students stated that media such as TV and newspaper were the most important factors influencing their decision to donate organs after brain death.

The students stated that media are mainly involved in causing the feeling of suspicion to death and thinking about organ donation. They caused a pessimism and distrust in the family toward the healthcare team due to watching movies that show selling organs and the possibility of getting alive after death.

One of participant said:

“Movies and TV series has shown that the brain-dead patient come back to life; perhaps one of them claimed so.” (Human sciences, 35 year old, male).

“I have watched several movies and video-clips about patients receiving organs from the brain-dead patients, and am impressed. Such movies have influenced my decision for organ donation.” (Medical sciences, 22 year old, female).

Most students were aware of the irreversibility brain death and organ donation. They said that one of the important factors influencing their decision was to have previous knowledge about the crisis of brain death and the phenomenon of brain death. Knowledge of this issue affected a voluntary decision to donate the organ.

The students said that even in case of having an organ donation membership card, the family satisfaction is still needed. People need to be sure about the correctness of their decision. The manner of dealing with this condition and how to inform people can influence their decision making.

“I heard about brain death in which the person is really dead, but the family must know that the person never comes to life again” (Human sciences, 30 year old, female).

The experience of individuals and their families regarding organ donation, knowing someone who needed organ, information about the life situation of patients needing organ transplantation may have negative or positive effects on their decisions for organ donation.

“If I change my mind and decide to donate my organs, I think I only donate my kidneys, because some of my relatives suffer from kidney failure, and it can extend their life.” (Medical sciences, 35 year old, female).

“If someone needs organ transplantation urgently, and he/she is waiting for my decision, I would probably do that.” (Technology and engineering, 26 year old, male).

### Theme 2: Mediators of decision making

This theme encompassed people, events, and so on that mediated the effect of other factors in making a decision for or against organ donations. This theme was consisted of two categories as the characteristics of the brain-dead patient and his/her family consent.

#### The consent of the brain-dead patient

Of the students who agreed to donate organ, the implicit or explicit consent of the brain-dead patient was of high importance. Those students who were against organ donation did not consider it important. Their perspectives have been provided as follow:

“Of my family members, my oldest daughter who lives in the USA has the organ donation card. I severely have cried for donating her organs after brain death, but I would not prevent her from donating her organs, because it is what she wants to.” (Medical sciences, 54 year old, female).

“If someone has an organ donation card, and fills out the associated forms, and tells his/her decision to his/her family member, this is okay, if not, I disagree to donate his/her organs, because if he/she wanted to donate his/her organs she/he should have had at least an organ donation card.” (Medical sciences, 30 year old, female).

#### The effect of family consent

Those students who agreed or disagreed with organ donation explained that they would agree to donate the organs to their relatives after brain death, only if their families agreed to do so.

Many students expressed the influence of the interaction between different family members and the effect of this interaction on the family’s decisions. The involvement of family members in the process of donation played an important role in their satisfaction with donation, despite their initial opposition.

“It is a hard to make a decision. I cannot tell you now that I would be disagree to donate the organs of my relatives who are brain dead. I am more certain if the brain-dead person is me rather than my relatives. It depends on the decision of my family, I would agree with what they decide to do.” (Technology and engineering, 25 year old, female).

### Theme 3: Beliefs and motivations

This theme was contained of two categories as internal beliefs and motivations, and religious beliefs.

#### Internal beliefs and motivations

This category had two sub-categories as humanitarian approach and personal beliefs.

#### Humanitarian approach

This sub-category included altruism, giving the life to others who need organ transplantation, helping people who urgently need an organ transplantation, alleviating others’ suffering, which were explained by those students who were agreed to organ donation. Quotations were reported as follow:

“I think that when I am able to help others to alleviate their suffering, organ donation is the best idea, because I am dead and my organs will be buried with me.” (Human sciences, 23 year old, male).

“My answer to organ donation is positive, because I believe that all humans must do it, saving the life of other people is important.” (Technology and engineering, 23 year old, male).

Personal beliefs, believing in forgiveness, contributing to the advancement of science, and having a good memory of the brain-dead person, were some personal beliefs positively associated with organ donation. Whereas, suffering the soul after organ donation was a negative personal belief associated with organ donation.

“I believe that organ donation contributes to the advancement of science. Through donating the organs of brain-dead patients we help find new methods for surviving the human being.” (Medical sciences, 54 year old, female).

“I believe that the soul remains alive after death and watches the organ donation scene as this causes the soul to suffer.” (Technology and engineering, 28 year old, female).

#### Religious beliefs

This category had two categories as religious belief encourages people to donate their organs and religious beliefs prevents people to donate their organs.

#### Religious beliefs encourages people to donate their organs

Religious beliefs play an important role in making the decision regarding organ donation. Believing in another life after death, forgiveness of guilt, and the soul peace were some religion aspects described by the students.

“Organ donation is the right thing to do, as death is not the end of life, and after death I begin another life.” (Technology and engineering, 28 year old, male).

“God may forgive my faults, because of organ donation and bring peace to my soul.” (Human sciences, 24 year old, female).

#### Religious beliefs prevent people from donating their organs

There are some religious factors that prevented the students from donating their organs. For instance, organ donation was in contradiction with bodily resurrection, the sins of organ donating person might be transferred to the organ receiving person, it was not worthy to save the life of some people, organ receiving persons normally do not have a high quality life, our fate was written before, unwillingness to mixing up bodies, and the family of the person who received the organ always was under stress.

“I think my body will be incomplete in the Doomsday.” (Technology and engineering, 23 year old, female).

Some person believed that organ donation was an interference in the God’s affairs.

“I do not know anything about the person who receives my body organs, what if he/she is a bad guy and it is an interference in God’s affairs.” (Medical sciences, 45 year old, female).

“I think that organ donation is a cause of defect in the body, cause a torment of the individual soul. Those organs by which a person has lived with them for a longer time, gets unavailable” (Technology and Engineering, 25 year old, Male).

“I feel that my body is dumb on the resurrection day and maybe somehow I have oppressed my body. I think it was a kind of organ defect for my body”. (Human sciences, 34 year old, Female).

### Theme 4: Interactions with the health system

This theme was consisted of two categories as medical errors and a lack of trust in equipment in the country. Medical errors had three sub categories as fear that physicians made a wrong diagnosis about brain death, a lack of competent specialists and sufficient equipment in the country, and a lack of trust to the medical system. The lack of trust to the healthcare services had three subcategories as the lack of a system for supervising organ donation and transplantation in the country, not giving enough time to families to make a decision, and lack of a distinct organization for protecting organs from being misused.

### Medical errors

Some students said that aggression for donation means that the hospital staff may not do their best efforts to save the patient. They liked to have a trusted doctor to confirm their diagnosis before donation. They also said that brain-dead patients sometimes transmitted from one province to another, and that there was no strict monitoring on organs transplantations. Also, while paying money for organ donation is legal in Iran, so there is a possibility of abuse, and rich and famous people may be placed at the top of the waiting list for transplantation.

“My sister has told me that sometimes physician makes a wrong diagnosis about brain death, there always is a possibility of wrong diagnosis.” (Human sciences, 26 year old, female).

“I always hear that some physicians are unable to diagnose appropriately, what if they make a wrong diagnosis about brain death? There are a few component specialists in Iran.” (Technology and engineering, 26 year old, male).

“I am not sure that donating organs will be received by those who really are on the waiting list, as they may be sold to rich people with a higher price.” (Medical sciences, 38 year old, female).

### The lack of trust to the health care services

Students believed that the structure of organ donation in Iran was not correct. Transplantation units affiliated with universities in Iran were very limited, and many of them were not functioning properly. It is not clear who is responsible for monitoring the appropriate implementation of this law by these units. For this reason, doctors were not subject to criminal prosecutions in the event of medical misconduct. Accordingly, doctors and nurses did not receive proper education and did not fully understand the timing of the proposal of organ donation to families.

“I do not have the organ donation card, I have tried twice for online registration, both times the web page was disconnected and I failed to register. I do not know where I can register.” (Medical sciences, 34 year old, female).

“If there is a misdemeanor at the time of donation, for example, I just agree that only my kidneys are donated not other organs, what will happen if my wills are not taken care by physicians. Who will follow this care?” (Technology and engineering, 26 year old, male).

“The person who has lost a loved one is plagued enough. Now, it seems to me that it is not right to put pressure on him. Many times, the person is talked and put under pressure to give the consent, and I do not think it is rational.” (Medical sciences, 30 year old, female).

### Theme 5: Dependency

This theme included two categories as surrender and acceptance.

The students said that being in a critical condition for the death of loved ones lead to a lot of psychological problems in them. Therefore, most students because of the severe emotional attachment to their family members disagreed with organ donations in case of brain death. Therefore, they stated that preparing the society to face the crisis of brain death of their loved ones could facilitate to encounter such a condition. In their views, as the crisis progressed, people’s perspectives would become more logical regarding organ donation.

#### Acceptance

Acceptance included sadness about the relatives who became brain-dead and were willingness for donating their organs.

“It is difficult to admit that my mother suffered from a brain death and I want to donate her members, but since she has showed her willingness to organ donation, I will surely donate her organs and do not allow them to be buried and remain useless.” (Medical sciences, 31 year old, female).

#### Surrender

Surrender had two categories as troublesomeness and disagreement. Troublesomeness meant that people did not tend to think about brain death in their relatives.

“I do not like to talk about it; it is easier to talk about brain death of myself rather than my relatives.” (Human sciences, 26 year old, female).

Disagreement was consisted of those cases that severe emotions did not let them to donate their organs.

“It is hard for my family, relatives and my friends to see the slaughtering of my body after death. I think my family cannot afford it. If I will be there I will definitely say no.” (Medical sciences, 34 year old, female).

### Theme 6: body integration

This theme was composed of two categories as the fear of being slaughtered and the feeling of being guilty.

The students’ idea of organ donation was that they themselves or one of their family members needed to undergo a surgical incision, and their so-called body was torn and frayed, which caused them to be afraid of organ donation.

The discussion of fear and visualization of the above-mentioned incident are a kind of human sense that exists in everyone. It is hard to imagine having a wound on ours or our loved one bodies. In the beliefs of the Iranian people, it is preferable that the body is brought to the soil, because it is believed that their dear lost is a part of their existence.

#### Fear of being slaughtered

“I think after death, the slaughtering of the human body is not tolerable and it is disgusting.” (Technology and engineering, 22 year old, female).

“It is hard to see that the body of my child is slaughtered, as I can accept the death of my child, but seeing him/her being slaughtered is not possible for me. It is a fight against my senses and emotions, but I can afford it.” (Medical sciences, 54 year old, female).

#### Feeling of being guilty

“I think organ donation makes the human being to be mutilated, which causes a feeling of being guilty.” (Technology and engineering, 22 year old, male).

## Discussion

The most important factor influencing the students’ attitudes of donation of their organs were cognitive readiness, mediators of decision making, beliefs and motivations, interactions with the healthcare system, dependency, and body cohesion.

The results of the present study were in line with those reported by other studies. For instance, Manzari et al. identified six main categories regarding decision for organ donation including the acceptance of brain death, cognitive readiness, quality and time of exposure, mediators of decision making, family interactions, and beliefs and motivations. Whereas, they did not mention dependency, body cohesion, interactions with the healthcare system, the feeling of being guilty, luck of trust to the healthcare system, and impact of family consent [21].

Knox in a systematic review stated that demographic characteristics, knowledge, attitude, and belief were main factors affecting the family decisions regarding organ donation [31].

Most participants in this study agreed with organ donation in the event of brain death. It could be due to their age, education level, and awareness of brain death and their relationships with a positive attitude toward organ donation [8, 10, 26, 32]. Attitude is one of the determinants of behavior and is influenced by the individual’s awareness and feeling. Studies have shown that if people become are aware of brain death, they have a more positive attitude toward organ donation. People with higher levels of awareness have more positive attitudes and more social trust to organ donation. These factors create the necessary motivation for organ donation in individuals. While the general public in Iran considers brain death and coma the same phenomenon and reversible recurrence, and therefore refuses to organ donation [20]. Students in this study had enough knowledge about brain death, and well recognized the difference in brain death from coma and pointed to its irreversible identity.

Educated people have a more positive attitude toward organ donation due to higher awareness of brain death and organ donation. In this study, “the contribution to the development of medical science and respect for the individual at the time of his/her life” was very important for each student. While the “reversibility of the patient after brain death” was not important [25]. Also, young people have a high desire to organ donation due to their personality traits and to benefit more from this capital, it is better to promote a culture of organ donation in an early age. In the present study, the average age of participants was 30.4 years [33].

In Iran, young people and especially students are influenced by Western culture and their lifestyles are more likely to be similar to that. Thus, an attitude toward organ donation that could be a taboo or unacceptable in the parent’s cultures such as the fear of selling the organ, slander of the corpse and the fragmentation of the body, were not available in their perspectives [34].

Considering that education is convertible into economic and social capital through the acquisition of jobs with good social status. Educated people can become models for the society in the future [35]. Therefore, the higher the attitude of these individuals toward organ donation, the higher effect on the improvement of the community’s attitude toward organ donation. On the other hand, it is possible to use the key role of student in Iranian families.

The interaction of family members with each another affects their decisions, such as donate organs at the time of brain death [21, 27]. The involvement of family members in the process of requesting organ donation plays an important role in the satisfaction of family members, despite their initial opposition. The selection of the appropriate person for presenting the organ donation request has an important role in gaining family trust to the treatment team and thinking positively about organ donation [21]. Therefore, if student with a high awareness and a positive attitude toward organ donation is asked to attract the family’s consent, it will be a great resource.

Due to their age and social status, students have a high degree of activity and are creative. Given the effective role of the university in expanding the society culture, students can play an effective role in cultural fields [36]. Ambassadors for organ donation are one of the most important aspect of culture development across the world [37]. Considering the students’ positive attitude, they can use volunteer students to become volunteer ambassadors for informing others.

The findings about the cognitive readiness theme was in line with those of i’Alessandro et al. [38] and Ralph’s et.al [12] studies. A lack of understanding regarding brain death affected the decision by individuals against organ donation. In the present study, awareness, previous experience, and media were categorized under the cognitive readiness theme which was consistent with those of other studies [21, 39].

The role of brain-dead patient was reported by other studies with the highest influence on the family decision [40, 41], which was not in line with the results of this study, in the present study, it was found as a category under the theme of “mediators of decision making”.

Religious beliefs in this study were placed under the category of “beliefs and motivations”. Although many studies have been conducted around the world on Muslims, religious beliefs are major factors influencing the Muslims’ lack of willingness to organ donation [42–44]. Most students participating in this study had a positive attitude toward organ donation, and only 9 people did not tend to donate organs due to religious issues.

Many Muslim scholars consider organ donation from a dead person legitimate, when there is an emergency. However, conditions have been laid down for transplantation including the demonstration of brain death and its irreversibility, the declaration of the individual’s consent to this action during the lifetime, the consent of his or her legal guardians and consideration of respect to the dead [45].

In Iran, Shi’a scholars also endorse organ donation based on the above- mentioned conditions. Therefore, in Iran, the process of organ donation is such that after confirming brain death, the subject of the donation is offered to the patient’s family. If they are satisfied, the consent form will be signed by them. In spite of having an organ donation card, the donation cannot take place in the absence of the family consent [46].

Therefore, the difference in Muslim tendencies in Iran compared to Muslims in Western countries can be related to policies and laws in these countries, which has led Muslims less willing to donate organs. In many countries such as the UK and the EU countries (Austria, Spain, Portugal, Austria), there are laws on consent assurance. If a person does not want to donate organs, he/she must declare his/her opposition before death. Otherwise, if a brain death occurs, transplanted organs must be removed [47]. The survey conducted in the United Kingdom and the United States on Muslims showed that they were strongly opposed to the laws of consent assurance [45, 48].

Another reason could be cultural differences. Iranian culture strongly emphasizes altruism, sacrifice, and helping one another. Young people and students are heavily influenced by Western culture. The Gauher’s et al. study also showed that young British-Pakistani population in Britain felt that, as the British culture was accepted (where donation was more acceptable), the attitude for organ donation by their parent’s cultures was unacceptable and should be ignored [44]. This reflected the profound effect of culture on organ donation. Also, the participants in the Gauher’s et al. study similar to the students participating in this study, emphasized the resilience of religion and its importance for generations [44]. The findings showed that the young Iranian population had a different perspective to religion compared to previous generations and had different perspectives about Islamic doctrine.

Whereas, Sohal et al. in a study in the Netherlands concluded that religious beliefs were not so important for making a decision for or against organ donation. The role of religious beliefs is more predominant among Muslims and Asian populations [23].

In the Gauher’s et al. study, Pakistani participants emphasized that they tend to act in accordance with their religion, and the lack of a definitive rules on the eligibility of organ donation in Islam reduced the likelihood of organ donation in Pakistan [44]. While many Islamic scholars believed that Islam permitted organ donation [49]. However, this message has not had a dominant influence over the young Muslim population of Pakistani living in England [44]. Gauher et al. stated that if rules that allowed organ donation were approved, it would be highly beneficial for a positive attitude toward organ donation [44].

While in Iran all Shiite scholars have consensus on the permissibility of donating organs. Due to the recent cultural work done in the media and numerous social networks, it has been well established in the Iranian society.

Siminoff et al. [50] explained that the cultural position, mental and psychological factors, beliefs, and dependency of persons on their family affected the tendency of people about organ donation. They were attributable to the theme of dependency found in the present study. The findings were also in line with those reported by Hosein Rezaei et al. [51].

The results of this study showed that despite the fact that most participants in the study agreed with organ donations at the time of brain death, and having proper information about brain death and accepting irreversibility after brain death, due to the severe emotional attachment to the family, disagreed with organ donations in the event of brain death. Many participants were not even willing to think about brain death and donations of their loved one organs. For various reasons, they were not willing to accept the reality of death. This suggests that the concept of brain death for all people with any amount of knowledge about brain death and organ donation is largely contradictory and ambiguous. Facing with the diagnosis of brain death of loved ones for the family means entering a space full of challenges, conflicts, ambiguities and conflicts, which becomes more complicated with sadness and psychological defensive reactions. The Manzari’s et al. study also showed the same results [20]. Considering that in Iran, the most important factor influencing the success of organ donation is a lack of consent by the family, the acceptance of brain death by the patient’s family plays a major role in organ donation [21].

A lack of tendency of family members to organ donation considering the appropriate information about brain death, could be due to their subjective background of organ donation. Therefore, one of the important issues in the placement of organ donation culture is that a family member has a subjective background when he/she is faced with a donation request to prevent negative feedbacks toward the donation process.

On the other hand, studies have shown that there is a relationship between understanding brain death and the time of request for organ donations and how this request is addressed [52]. Therefore, nurses and physicians should have required knowledge on reporting brain death news and giving requests to family members regarding organ donation [20].

This study demonstrated that regardless of the motivations and characteristics of societies, populations were under the influence of different cultural and religious backgrounds and sociological characteristics. Many factors influence students’ decisions for or against organ donation including cognitive readiness, mediators of decision making, beliefs and motivations, interactions with the healthcare system, dependency and body cohesion.

To the best of our knowledge, the present study was the first study on students’ attitudes toward organ donation in Hamadan city, Iran. However, this should be regarded in the context of the methodological strengths and limitations of the study. The strengths of the present study was the maximum variance in sampling that enhanced conformability, credibility, and transferability of the study.

The limited sample size and having a specific sampling population of academic individuals hinders its transferability to the whole society. Future studies can be conducted using a quantitative method to be able to predict the effect of various factors on individuals’ attitudes about organ donation.

## Conclusion

The present study helped identify important factors regarding the organ donation decision. Identification and explanation of such factors are of pivotal as they can guide policy makers and managers to design interventions to promote the attitude and culture of the society regarding organ donation. The findings of the present study can be used to develop a tool for measuring the quantitative effect of each factor on organ donation.
